# New *Mycobacterium tuberculosis* Complex Sublineage, Brazzaville, Congo

**DOI:** 10.3201/eid2303.160679

**Published:** 2017-03

**Authors:** Sven Malm, Laure S. Ghoma Linguissi, Emmanuel M. Tekwu, Jeannhey C. Vouvoungui, Thomas A. Kohl, Patrick Beckert, Anissa Sidibe, Sabine Rüsch-Gerdes, Igor K. Madzou-Laboum, Sylvie Kwedi, Véronique Penlap Beng, Matthias Frank, Francine Ntoumi, Stefan Niemann

**Affiliations:** Leibniz Center for Medicine and Biosciences, Borstel, Germany (S. Malm, T.A. Kohl, P. Beckert, S. Rüsch-Gerdes, S. Niemann);; Fondation Congolaise pour la Recherche Médicale, Brazzaville, Congo (L.S. Ghoma Linguissi, J.C. Vouvoungui, A. Sidibe, S. Kwedi, F. Ntoumi);; University Marien Ngouabi, Brazzaville (L.S. Ghoma Linguissi, J.C. Vouvoungui, A. Sidibe, F. Ntoumi);; University of Yaoundé I, Yaoundé, Cameroon (E.M. Tekwu, V. Penlap Beng);; Institute for Tropical Medicine, University of Tübingen, Tübingen, Germany (E.M. Tekwu, M. Frank, F. Ntoumi);; Centre Antituberculeux de Brazzaville, Brazzaville (I.K. Madzou-Laboum);; German Center for Infection Research, Tübingen Site, Tübingen (M. Frank);; German Center for Infection Research, Borstel Site, Borstel (S. Niemann)

**Keywords:** Genetic diversity, Mycobacterium tuberculosis, transmission, phylogeny, epidemiology, Brazzaville, Congo, tuberculosis and other mycobacteria, bacteria

## Abstract

Tuberculosis is a leading cause of illness and death in Congo. No data are available about the population structure and transmission dynamics of the *Mycobacterium tuberculosis* complex strains prevalent in this central Africa country. On the basis of single-nucleotide polymorphisms detected by whole-genome sequencing, we phylogenetically characterized 74 MTBC isolates from Brazzaville, the capital of Congo. The diversity of the study population was high; most strains belonged to the Euro-American lineage, which split into Latin American Mediterranean, Uganda I, Uganda II, Haarlem, X type, and a new dominant sublineage named Congo type (n = 26). Thirty strains were grouped in 5 clusters (each within 12 single-nucleotide polymorphisms), from which 23 belonged to the Congo type. High cluster rates and low genomic diversity indicate recent emergence and transmission of the Congo type, a new Euro-American sublineage of MTBC.

Despite the availability of antituberculous drugs for the past 60 years, tuberculosis (TB) remains a major health threat worldwide. In 2014, the World Health Organization registered 6 million new TB cases, and 1.5 million affected persons died of infection with *Mycobacterium tuberculosis* complex (MTBC) strains, the causative agent of TB (*1*). Congo (Republic of Congo), in Central Africa, has a population of ≈4 million inhabitants and is considered to be a high TB incidence area; incidence is 381 cases/100,000 inhabitants (*1*). Approximately one fourth of the population agglomerates in its capital city, Brazzaville.

Despite the serious situation, precise data on disease dynamics and recent transmission patterns guided by modern molecular epidemiologic tools are only sparsely available. Molecular epidemiology is useful for analyzing MTBC strain diversity and transmission dynamics in low- and high-incidence settings (*2**,**3*). Furthermore, molecular typing has shown that MTBC has a diverse population structure with manifold lineages that show large differences in geography and pathobiological properties, such as the development and spread of drug resistance (*4**,**5*).

To address current knowledge gaps, we determined the population structure of MTBC isolates from patients with pulmonary TB in Brazzaville. Samples were collected from patients at the Centre Antituberculeux de Brazzaville during February–June 2011 (*6*). We investigated the population structure and transmission patterns by a combination of classical genotyping and whole-genome sequencing (WGS). Single-nucleotide polymorphisms (SNPs) detected by WGS were used for phylogenetic lineage classification and similarity analysis estimating recent transmission rates. This approach enabled detailed insight into the population structure and phylogeny of MTBC strains circulating in Brazzaville. Moreover, we describe a new predominant sublineage, the Congo type, which most likely forms a larger transmission network in the study area.

## Methods

### Study Design

The patient population was reported previously (*6*). In brief, 775 consecutive patients seeking care at the Centre Antituberculeux de Brazzaville during February–June 2011 were evaluated for pulmonary TB according to the national diagnostic algorithm (*6*). The institutional ethics committee, Comité d’Ethique pour la Recherche Scientifique, in August 2010 (no. 00000067/DGRST/CERSSA) approved the study. Informed consent for study participation, as well as permission to use isolates from samples provided, were obtained from all enrolled participants. Samples with the highest Ziehl-Neelsen score (semiquantitatively classified in categories 1+, 2+, or 3+ in on-site laboratories based on microscopic findings) out of at least 2 positive sputum samples from 1 patient (n = 211) were shipped to the Research Center Borstel (Borstel, Germany) for culture, drug susceptibility testing, genotyping, and WGS.

### Sample Processing, Culture, and Drug Susceptibility Testing

Approximately 5 mL of each specimen was homogenized by digestion for 1 min at room temperature with 1 mL of N-acetyl L-cysteine (25 mg/mL) in phosphate buffer (pH 6.8) and vortexed with several 4-mm glass beads for 30 s. A 5-mL aliquot was decontaminated by using 1% NaOH and concentrated at 4,000 × *g* for 15 min. The sediment was then reconstituted to 2.5 mL by using phosphate buffer pH 6.8 to make the inoculum for smears and cultures. Sputum was cultured by using the conventional Löwenstein-Jensen growth medium followed by determination of mycobacterial species according to standard techniques (*7*). Samples for susceptibility testing of first-line drugs were processed as described previously (*8*). Drug susceptibility testing was performed by using the BACTEC MGIT system (Becton, Dickinson and Company, Franklin Lakes, NJ, USA). Samples without antimicrobial drugs served as growth controls. Genomic DNA was extracted from sputum cultures on Löwenstein-Jensen medium, by using a standard cetyltrimethylammonium bromide–NaCl method (*3*).

### Traditional Genotyping

We performed spacer oligonucleotide typing (spoligotyping) as described by Kamerbeek et al. (*9*). PCR-negative controls were included in which distilled, diethyl pyrocarbonate-treated H_2_O was added instead of DNA. Genomic DNA of *M. tuberculosis* H37Rv and *M. bovis* BCG were included as controls. In addition to spoligotyping (*10*), we conducted mycobacterial interspersed repetitive unit–variable number tandem repeat (MIRU-VNTR) typing based on 24 loci as described previously (*11*); both 24-loci MIRU-VNTR typing and spoligotyping data analysis was performed by using the tools implemented at the MIRU-VNTRplus website (*12*). Genomic DNA of *M. tuberculosis* H37Rv was included as a positive control. We used the MIRU-VNTRplus nomenclature server (*11*) in addition to the definition of shared spoligotypes to assign a unique MTBC 15-9 number to each 24-loci MIRU-VNTR combination. Data were analyzed further only if all controls showed the expected outcome. All traditional genotyping techniques were performed at the Research Center Borstel, Leibniz Center for Medicine and Biosciences (Borstel, Germany).

### WGS and Data Analysis

We prepared libraries for next-generation sequencing from genomic DNA by using the Nextera XT library preparation kit and run with Illumina-supplied reagent kits on the HiSeq and MiSeq systems (Illumina, San Diego, CA, USA), according to the manufacturer’s recommendations. For 1 isolate (9679-00), genomic DNA was sequenced by GATC Biotech AG (Konstanz, Germany). WGS of the strains of the study population was conducted at the Research Center Borstel, Leibniz Center for Medicine and Biosciences. NGS data of sequenced isolates was submitted to the EMBL-EBI ENA sequence read archive (PRJEB9545).

We mapped sequence reads to the *M. tuberculosis* H37Rv genome (GenBank accession no. NC_000962.3) with the SARUMAN exact alignment tool (*13*). Genomic coverage was at least 50-fold for all isolates. Customized Perl scripts were used to extract SNPs from mapped reads, requiring a minimum coverage of 10 reads and a minimum allele frequency of 75% as detection thresholds (*14*). We excluded SNPs in resistance-mediating genes and repetitive regions from the phylogenetic analysis (*15*). Moreover, to avoid calling SNPs because of indel-related artifacts, we excluded SNPs within ± 12 nt from each other (*16*). Positions that matched these thresholds in all isolates were considered as valid and used for a concatenated sequence alignment.

We then calculated a pairwise distance matrix from concatenated SNP positions by Perl scripts, with +1 distance between paired isolates for each mismatching base, and plotted data in GraphPad Prism 5 (GraphPad Software Inc., La Jolla, CA, USA). On the basis of the distance matrix, we grouped isolates into putative transmission networks by incrementally accumulating all isolates with a maximum distance of 5 or 12 SNPs, respectively, to the nearest neighbor into 1 group (*16*). On the basis of the WGS data, we classified isolates into known phylogenetic groups according to the set of informative SNP positions published by Coll et al. (*17*).

We calculated the phylogenetic tree using the maximum-likelihood method and the general time reversible (GTR) substitution model, rate heterogeneity, without invariant sites using a gamma distribution as well as bootstrap resampling. Substitution models were tested and trees calculated by using MetaPiga software version 3.1 (*18*) and the maximum-likelihood ratio test (*19*). We applied mid-point rooting with FigTree software version 1.4.2 (http://tree.bio.ed.ac.uk/software/figtree/) and performed formatting by using the online tool Evolview (*20*). Specific SNPs for the Congo-type sublineage, and the more distant undefined strain 8095/11, were extracted by the ancestral states reconstruction method, implemented in the MetaPiga version 3.1 software, for both the specific and the common node. The maximum parsimony tree for visualizing genome-based clusters was calculated with Bionumerics 7.5 software (Applied Maths, Kortrijk, Belgium).

We used the concatenated sequence alignment in a Bayesian coalescent analysis with BEAST version 1.8.2 to infer node ages in the genealogic tree (*21*). A tip dating approach was not possible; therefore, we used a strict molecular clock prior of 1 × 10^−7^ substitutions per site per year and compared different demographic models and a GTR versus Hasegawa, Kishino and Yano substitution model using a chain length of 10 million and sampling of every 1,000 generations with a burn-in of 10% that resulted in adequate mixing of the Markov chains and effective sample sizes in the thousands. The comparison of the likelihoods of each run with Tracer version 1.5 showed very strong support of the GTR substitution model over Hasegawa, Kishino and Yano (log_10_ Bayes factors >93) and no preference for a particular demographic model; thus, we used a coalescent constant size model, representing the most straightforward approach. Resulting data were combined in a maximum clade credibility tree by using TreeAnnotator version 1.8.2 to infer node ages and highest posterior density intervals (*21*).

## Results

### Study Population

Results of sputum smear microscopy, radiographic abnormalities, and HIV infection were reported previously (*6*). In brief, 211 sputum samples of patients with suspected TB were sent to the National Reference Center for Mycobacteria (Borstel, Germany); 75 cultures yielded positive results. Phenotypic characterization identified 1 *M. intracellulare*, 6 *M. africanum*, and 68 *M. tuberculosis* isolates. We excluded the *M. intracellulare* isolate from further analysis. The mean age ± SD of the 74 TB patients was 33.86 ± 11.65; 69 (93%) patients were <50 years of age, and 66% were male. The patients’ residences were distributed in the different districts of Brazzaville as follows: southern part, 31% from Makélékélé and 4% from Bacongo; and northern part, 22% from Talangai, 8% from Poto-poto, and 7% from Ouenzé (4%). Of the 74 study participants, 13 (18%) were HIV co-infected.

### Drug Susceptibility Patterns

We determined phenotypic drug susceptibility patterns for the first-line anti-TB drugs isoniazid (INH), rifampin (RIF), ethambutol (EMB), and pyrazinamide (PZA) for all strains. In case resistance against 1 of these drugs was detected, streptomycin (STR) and second-line antimicrobial drugs were included in the analysis. Of the 74 MTBC strains, 71 (96%) were fully sensitive to all the first-line anti-TB drugs. Three (4%) isolates were resistant: 1 isolate was resistant against INH and STR, and 2 isolates exhibited a multidrug-resistant (MDR) phenotype with resistances against INH, RIF, PZA, and EMB (Table). The 2 MDR strains underwent susceptibility testing on second-line anti-TB drugs; 1 isolate was resistant to ethionamide. No extensively drug resistant strains were identified.

**Table:** Description of lineage and associated *rpoB*, *katG*, and *fabG1-InhA* mutation identifiers in drug-resistant *Mycobacterium tuberculosis* isolates, Congo*

Isolate code	Resistance	Gene	Nucleotide change	Amino acid substitutions	Lineage
8032/11	INH, STR	*katG*	AGC-315-ACC	Ser-315-Thr	Congo type
8114/11	INH, RIF, STR, EMB, PZA, ETH	*katG*	AGC-315-ACC	Ser-315-Thr	Uganda I
		*rpoB*	GAC-516-GTC	Asp-516-Val	
		*inhA*	−102G/A	NA	
8125/11	INH, RIF, STR, EMB, PZA	*katG*	AGC-315-ACC	Ser-315-Thr	Beijing
		*rpoB*	GAC-516-TAC	Asp −516- Tyr	

*EMB, ethambutol; ETH, ethionamide; INH, isoniazid; NA, not applicable; PZA, pyrazinamide; RIF, rifampin; STR, streptomycin.

### Population Structure of the MTBC Isolates

**Figure 1:**
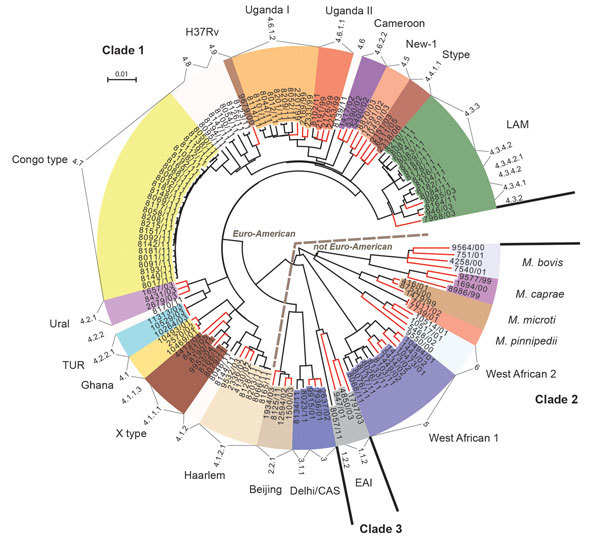
Maximum-likelihood tree of *Mycobacterium tuberculosis* complex isolates from Congo (black branch tips) and 65 reference strains (red branch tips). The tree was calculated by using the general time reversible substitution model with gamma distribution based on single-nucleotide polymorphisms identified by whole-genome sequencing. Models were tested and the tree generated by using MetaPiga software version 3.1 (*18*) and the maximum-likelihood ratio test. Midpoint rooting was performed. Distinct colors were chosen for the lineages identified; leaves with white background represent strains that initially were not assigned to particular lineages because of ambiguous typing patterns from mycobacterial interspersed repetitive unit, restriction fragment length polymorphism, or spoligo analysis (data not shown). The numerical code assigned to the respective lineages at the outer rim of the circular tree shows the Coll-nomenclature inferred from the whole-genome sequencing data. EAI, East African Indian; LAM, Latin American Mediterranean; TUR, Turkish. Scale bar indicates nucleotide substitutions per site.

**Figure 2:**
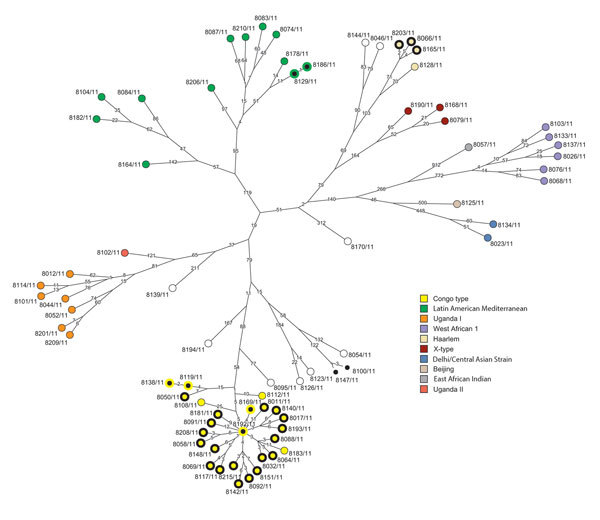
Maximum parsimony tree of *Mycobacterium tuberculosis* complex isolates from Congo. The tree was calculated on the basis of the concatenated single-nucleotide polymorphism (SNP) list. Branch labels indicate SNP distances; node labels represent the strain identifiers. Clusters based on pairwise comparisons were plotted on the tree: clusters <5 SNPs, nodes filled in black; clusters <12 SNPs, nodes with bold black outlines.

For all isolates, we successfully performed classical genotyping and WGS. Overall, we detected 18,059 SNP positions, which we used for further interrogations. On the basis of these analyses, we classified the *M. tuberculosis* strains into the main phylogenetic lineages Euro-American (n = 64); Delhi/Central Asian (n = 2; Coll lineage 3 and sublineage 3.1.1); Beijing (n = 1; Coll sublineage 2.1.1); East African Indian (n = 1; Coll sublineage 1.2.2); and *M. africanum* West African-1 (n = 6; Coll lineage 5). The Euro-American strains split into Latin American Mediterranean (LAM; n = 12; Coll sublineages 4.3.2, 4.3.4.1, 4.3.4.2, 4.3.4.2.1 and 4.3.3); Uganda I (n = 7; Coll sublineage 4.6.1.2); Uganda II (n = 1; Coll sublineage 4.6.1.1); Haarlem (n = 4; Coll sublineage 4.1.2.1); and X type (n = 3; Coll sublineage 4.1.1.1). Eleven Euro-American strains not well classified by MIRU-VNTR/spoligotyping (mainly T) belong to the Coll sublineages 4.1.2, 4.2.2, 4.6, 4.7, 4.8, and 4.9. Although few 4.7 strains were present in the collection analyzed by Coll et al. (*17*), they are dominant in our study collection and thus were termed Congo type (n = 26). These strains form a clear-cut branch in the MIRU/spoligotyping (data not shown) and SNP-based phylogeny (Figures 1, 2). Thus, these strains most likely represent a new Euro-American sublineage circulating in the region.

For an in-depth view on the population structure and to define the position of the Congo-type strains in the MTBC phylogeny, we analyzed the SNPs detected by WGS together with a set of reference strains (65 strains) previously used in the MIRU-VNTRplus dataset (*11*). Our reference collection comprises 3 clinical isolates of the major lineages of the MTBC and the type strains *M. tuberculosis* H37Rv ATCC 27294, *M. bovis* ATCC 19210, and *M. africanum* West African 2 ATCC 25420 (*22*). On the basis of the 18,059 SNP positions, we calculated a maximum-likelihood tree (Figure 1) that shows that the Congo-type strains cluster most closely with H37Rv, presumably being part of a larger sublineage of H37Rv-related strains mentioned in other settings (*23**,**24*). Seven additional strains belonging to the Coll lineages 4.8 and 4.9 form a specific branch together with Congo type and H37Rv. We generated a tree containing bootstrap values supporting the branches of the tree (Technical Appendix Figure 1).

Bayesian coalescent analysis approximated the last common ancestor of all Congo-type strains in our study to 1958 (95% highest posterior density 1947–1967). Thus, the Congo-type strains of this study probably emerged in the past 53 years.

WGS enabled us to identify SNPs specific for the Congo type by extracting the SNPs from the node specific for the Congo-type sublineage only and the common node for the Congo type and the more distant strain 8095/11. We found 49 SNPs unambiguously associated with the Congo-type sublineage (online Technical Appendix Table). The analysis of the pairwise distances revealed a homogenous population of the Congo-type strains with a median pairwise distance of 20 SNPs only (range 2–53 SNPs), whereas this distance was larger among the strains of other lineages (Technical Appendix Figure 2).

The MDR isolates belonged to the Uganda and the Beijing lineages. By contrast, the strain resistant against INH and STR could be assigned to the Congo-type sublineage. We found no significant association between the Congo type and basic variables, such as sex, age, date of specimen collection, or patients’ residence or HIV status.

### Cluster Analysis

Among the strains in the study population, we identified 4 groups of strains with pairwise distances within 0–5 SNPs. Such close relationships between the strains indicate recent transmission. Two of the genome clusters are formed by Congo-type strains (2 clusters formed by 2 strains, 15% of the Congo-type strains). The other 2 genome clusters consisted of 2 isolates each, of either the LAM or Coll sublineage 4.8 closely related to H37Rv.

When we used a wider cluster definition of 12 SNPs maximum distance, 30 strains were grouped in 5 clusters ranging in size from 2 to 20 strains (Figure 2). Overall, 23 of the 26 Congo-type strains (88.5%) are in 1 large (n = 20) and 1 smaller (n = 3) cluster. Three (75%) isolates of the Haarlem lineage and 2 (17%) isolates of the LAM lineage are grouped in such clusters.

## Discussion

Similar to other countries in Central Africa, Congo has a high incidence of TB. Our aim was to determine the population structure and transmission dynamics of the MTBC strains in Brazzaville.

Our genotyping approach showed that all strains investigated were either *M. tuberculosis* or *M. africanum*; *M. tuberculosis* was most prevalent. These findings are in accordance with most recent studies from the other African countries reporting a predominance of *M. tuberculosis* strains (*25**–**28*). The high prevalence of *M. tuberculosis* detected in our investigation suggests that this predominance of *M. tuberculosis* strains might equally be the case for Congo, mainly driven by the newly described Congo-type sublineage.

The closely related strains of the Euro-American Congo-type sublineage were responsible for 35% of TB cases in the study population and showed a low pairwise genetic distance resulting in a high genome-based cluster rate, indicating ongoing recent transmission. It is tempting to speculate that strains from the Congo type are highly successful in the area and are recently expanding in the region of Brazzaville. On the other hand, strains of other MTBC lineages showed a higher degree of genetic diversity and formed smaller clusters with <3 strains. Such a high diversity is somewhat unexpected because, in a TB-endemic area, only few dominant clones, such as the Congo type, are hypothesized to circulate (*29*). The aforementioned findings point to a particular capacity of strains of the Congo type to spread in the area; for example, because of adaptation to the host population, as already postulated for other MTBC lineages (*5**,**30*). Accordingly, the diversity in the other MTBC lineages might reflect a higher rate of cases from reactivation of past TB infections, as suggested by a study conducted in South Africa with a high incidence of TB and high strain diversity (*31*).

The dominance of particular highly spreading clones, however, appears to be a more general phenomenon seen in several high-incidence areas. For example, strains of the Beijing lineage dominate in East Asia (*5**,**32*); the F11 *M. tuberculosis* genotype in Western Cape, South Africa (*31*); the LAM10-Cam family in Cameroon (*25*); and the K family, a sublineage of the Beijing genotype, in South Korea (*33*). In focusing on Africa, recent investigations revealed that Cameroon MTBC strains are responsible for most TB cases in several West Africa countries, such as Ghana and Cameroon (*25**,**27*); strains of the Uganda sublineage predominate in East Africa (*34*); and strains of the Sierra Leone sublineage predominate in Sierra Leone (*35*). Together, these data indicate marked differences in circulating mycobacterial strains in different Central Africa countries, suggesting a region-specific selection and spread of dominant sublineages of the Euro-American lineage.

Genome analysis enables not only high-resolution description of MTBC population diversity but also improved resolution of strains in recent transmission chains (*14**,**16*). Consistently, a SNP distance up to 5 SNPs was found in strains from confirmed direct human-to-human transmission, whereas a 12-SNP distance was proposed as a threshold to define larger cluster/transmission networks (*14**,**16*). Using these thresholds, we found that strains of the Congo-type sublineage formed a genetically homogenous group with median pairwise genome SNP distances of 20 SNPs (range 2–53 SNPs) and a large number of Congo-type strains in clusters differing by ≤5 SNPs (15%) or a maximum of <12 SNPs (88.5%). This finding supports the presence of a larger transmission network of the Congo-type strains that presumably emerged in the past 53 years.

Only 2 of the 74 isolates characterized in this study were MDR, whereas recently Aubry et al. reported a higher MDR rate among MTBC isolates from Brazzaville and Pointe Noire (15 of 46 strains investigated; 10 strains belonging to the same lineage based on MIRU-VNTR and spoligotyping) (*36*). However, 7 of the 15 MDR strains in that study were obtained from retreated TB patients within a short 9-day sampling period. Even though we detected only 2 MDR strains of different lineages in the population in our study, the presence of MDR strains might pose a serious future challenge to public health authorities because these strains might have the potential to spread in the population; in line with this concern, 8 of the MDR strains described by Aubry et al. have been isolated from persons with newly diagnosed cases, which the authors suggested might reflect the transmission of an MDR clone (*36*). Thus, the development of drug-resistant phenotypes among particular lineages circulating in Congo, especially the successful Congo type, should be revisited as part of a larger investigation to evaluate the actual extent of MDR TB and potential pockets of transmission in Congo.

The data from our genome-based investigation of circulating MTBC strains in Congo demonstrate the presence of a new, predominant, and highly transmissible sublineage, the Congo type, which belongs to the Euro-American lineage. Larger molecular epidemiologic studies with respect to sociogeographic data and in addition to traditional contact tracing investigations will be required in Central Africa to gain a better understanding of recent transmission networks, the emergence of dominant lineages, and the prevalence of drug-resistant phenotypes in this wider geographic setting. Such studies may be implemented in large networks, such as the Central Africa Network on Tuberculosis, HIV/AIDS and Malaria (sponsored by the European and Developing Countries Clinical Trials Partnership), with the objective of conducting baseline investigations of TB in its members state: Gabon, Cameroon (*37**,**38*), and Congo (*6*).

Technical Appendix Single-nucleotide polymorphisms specific for the Congo type sublineage of *Mycobacterium tuberculosis* as determined by whole-genome sequencing; maximum-likelihood tree of the study population and 65 reference strains; and intralineage pairwise distance comparison of the Republic of the Congo study population.
