# Supplementary material for: New Mycobacterium tuberculosis Complex Sublineage, Brazzaville, Congo
Source: Emerg Infect Dis. 2017 Mar;23(3):423–9. doi: 10.3201/eid2303.160679 (PMC5382753; doi:10.3201/eid2303.160679)
Supplement: Technical Appendix — Single-nucleotide polymorphisms specific for the Congo type sublineage of Mycobacterium tuberculosis as determined by whole-genome sequencing; maximum-likelihood tree of the study population and 65 reference strains; and intralineage pairwise distance comparison of the Republic of the Congo study population. [file 16-0679-Techapp-s1.pdf]

Article DOI: http://dx.doi.org/10.3201/eid2303.160679

New *Mycobacterium tuberculosis* Complex Sublineage, Brazzaville, Congo

Technical Appendix

Technical Appendix Table. Single-nucleotide polymorphisms specific for the Congo type sublineage of *Mycobacterium tuberculosis* as determined by whole-genome sequencing*

No.	Node specific for Congo type and more distant isolate 8095/11	Node specific for Congo type	Consecutive no.	Position, genome	Referent	Gene	Annotation	Category
1	C	T	920	164043	C	Rv0136	Probable cytochrome P450 138 Cyp138	Nonessential
2	C	A	1714	324590	C	Rv0270	Probable fatty-acid-CoA ligase FadD2 (fatty-acid-CoA synthetase) (fatty-acid-CoA synthase)	Nonessential
3	C	T	3273	711500	C	–	–	–
4	G	A	3398	738234	G	–	–	–
5	T	G	3431	745650	T	Rv0648	Alpha-mannosidase	Nonessential
6	A	C	3703	808925	A	Rv0712	hypothetical protein	Nonessential
7	C	T	4048	896374	C	Rv0803	Phosphoribosylformylglycinamidine synthase II PurL (FGAM synthase II)	Essential
8	T	A	4380	987573	T	Rv0888	Probable exported protein	Nonessential
9	C	A	4529	1017952	C	Rv0913c	Possible dioxygenase	Nonessential
10	C	G	4533	1018202	C	Rv0913c	Possible dioxygenase	Nonessential
11	G	C	4649	1044201	G	Rv0935	Phosphate-transport integral membrane ABC transporter PstC1	Nonessential
12	T	C	4760	1071036	T	Rv0958	Possible magnesium chelatase	Nonessential
13	T	C	5127	1151473	T	Rv1028c	Probable sensor protein KdpD	Essential
14	G	A	5734	1312379	G	Rv1179c	hypothetical protein	Nonessential
15	C	T	5861	1343858	C	Rv1200	Probable conserved integral membrane transport protein	Nonessential
16	G	A	6095	1398400	G	Rv1251c	hypothetical protein	Nonessential
17	C	T	6162	1418430	C	–	–	–
18	A	C	6419	1479338	A	Rv1318c	Possible adenylate cyclase (ATP pyrophosphate-lyase) (adenylyl cyclase)	Nonessential
19	G	A	6563	1517644	G	Rv1350	Probable 3-oxoacyl-[acyl-carrier protein] reductase FabG2 (3-ketoacyl-acyl carrier protein reductase)	Essential
20	G	C	6830	1594356	G	Rv1420	Probable excinuclease ABC (subunit C-nuclease) UvrC	Essential
21	G	A	7854	1860873	G	Rv1650	Probable phenylalanyl-tRNA synthetase, β chain PheT	Essential
22	C	T	8594	2066345	C	Rv1820	Probable acetolactate synthase IlvG (acetohydroxy-acid synthase)(ALS)	Nonessential
23	G	A	9066	2157937	G	Rv1911c	Probable lipoprotein LppC	Nonessential

No.	Node specific for Congo type and more distant isolate 8095/11	Node specific for Congo type	Consecutive no.	Position, genome	Referent	Gene	Annotation	Category
24	G	A	9846	2364826	G	–	–	–
25	G	A	9887	2377069	G	Rv2116	Conserved lipoprotein LppK	Nonessential
26	G	A	11029	2665887	G	Rv2380c	Peptide synthetase MbtE (peptide synthase)	Nonessential
27	G	A	11054	2672087	G	Rv2383c	Phenyloxazoline synthase MbtB (phenyloxazoline synthetase)	Nonessential
28	G	T	11067	2675026	G	Rv2383c	Phenyloxazoline synthase MbtB (phenyloxazoline synthetase)	Nonessential
29	C	G	11622	2819145	C	Rv2504c	Probable succinyl-CoA:3-ketoacid-coenzyme A transferase (α subunit) ScoA (3-oxo acid:CoA transferase) (OXCT A) (succinyl-CoA:3-oxoacid-coenzyme A transferase)	Nonessential
30	C	T	11642	2822369	C	–	–	–
31	G	A	11990	2894791	G	Rv2570	hypothetical protein	Nonessential
32	C	A	12333	2990901	C	Rv2674	Probable peptide methionine sulfoxide reductase MsrB (protein-methionine-R-oxide reductase) (peptide met(O) reductase)	Nonessential
33	G	A	12821	3105264	G	Rv2796c	Probable conserved lipoprotein LppV	Nonessential
34	G	A	13556	3284774	G	Rv2941	Fatty-acid-AMP ligase FadD28 (fatty-acid-AMP synthetase) (fatty-acid-AMP synthase)	Nonessential
35	G	C	13573	3291713	G	Rv2946c	Probable polyketide synthase Pks1	Nonessential
36	C	T	14174	3439813	C	Rv3077	Possible hydrolase	Nonessential
37	C	T	14276	3466263	C	Rv3097c	PE-PGRS family protein, triacylglycerol lipase LipY (esterase/lipase) (triglyceride lipase) (tributyrase)	Nonessential
38	G	A	14842	3606267	G	Rv3229c	Possible linoleoyl-CoA desaturase (delta(6)-desaturase)	Essential
39	G	A	14918	3620820	G	Rv3241c	hypothetical protein	Nonessential
40	G	A	15194	3687702	G	Rv3302c	Probable glycerol-3-phosphate dehydrogenase GlpD2	Nonessential
41	G	A	15560	3810807	G	Rv3394c	hypothetical protein	Nonessential
42	G	A	16005	3954731	G	Rv3518c	Probable cytochrome P450 monooxygenase 142 Cyp142	Nonessential
43	A	G	16295	4022055	A	Rv3579c	Possible tRNA/rRNA methyltransferase	Essential
44	G	T	16530	4082798	G	–	–	–
45	A	G	16543	4086449	A	Rv3646c	DNA topoisomerase I TopA (omega-protein) (relaxing enzyme) (untwisting enzyme) (swivelase) (type I DNA topoisomerase) (nicking-closing enzyme) (TOPO I)	Essential
46	G	A	16554	4088339	G	Rv3648c	Probable cold shock protein A CspA	Essential
47	G	A	17343	4267992	G	Rv3805c	Possible arabinofuranosyltransferase AftB	Essential
48	C	T	17569	4310863	C	–	–	–
49	C	G	17886	4391871	C	Rv3907c	Probable poly(A) polymerase PcnA (polynucleotide adenylyltransferase) (NTP polymerase) (RNA adenylating enzyme) (poly(A) polymerase)	Essential

*Dashes indicate no gene is assigned to this particular genomic positions.

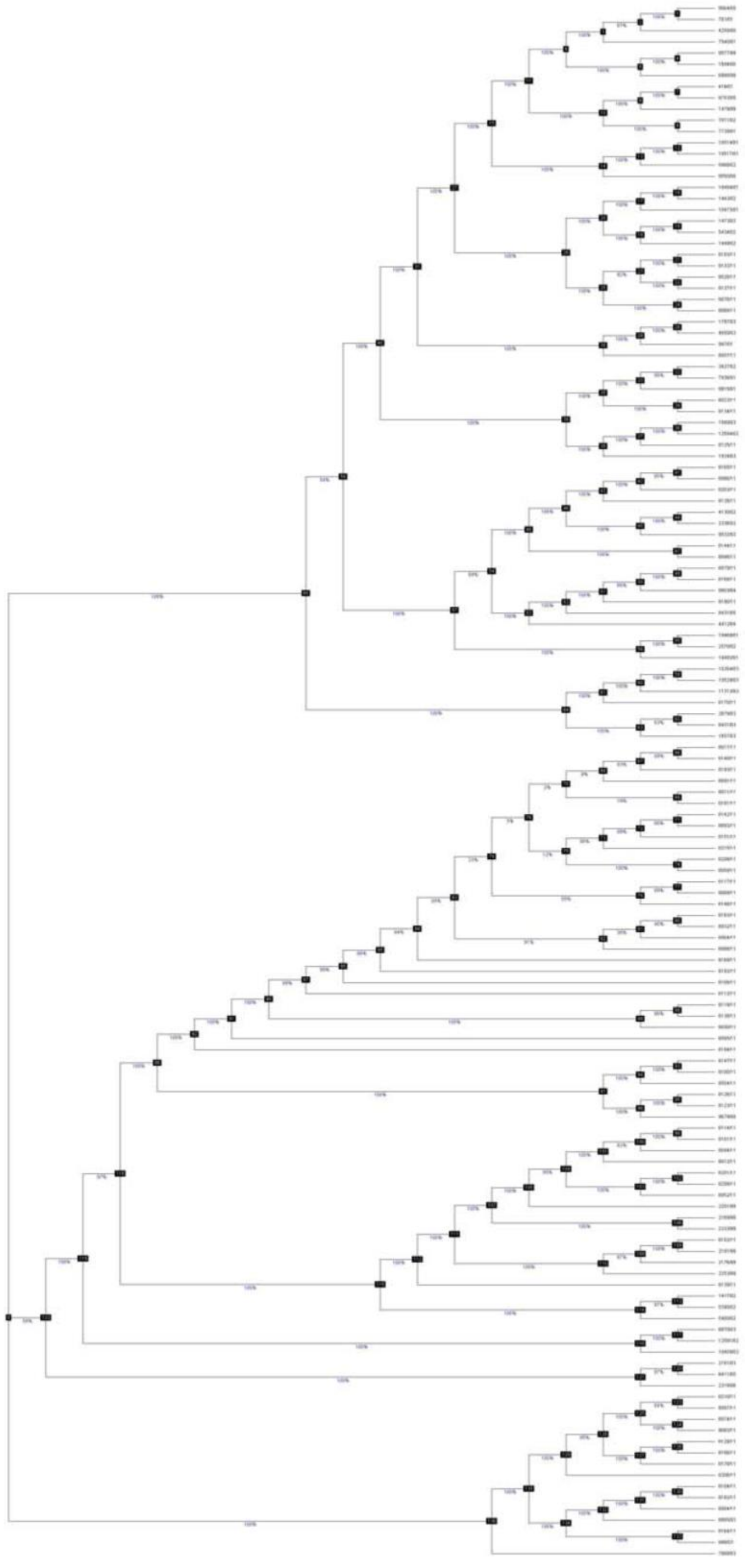

Technical Appendix Figure 1. Maximum-likelihood tree of the population in the Republic of the Congo *Mycobacterium tuberculosis* study and 65 reference strains showing supporting bootstrap values.

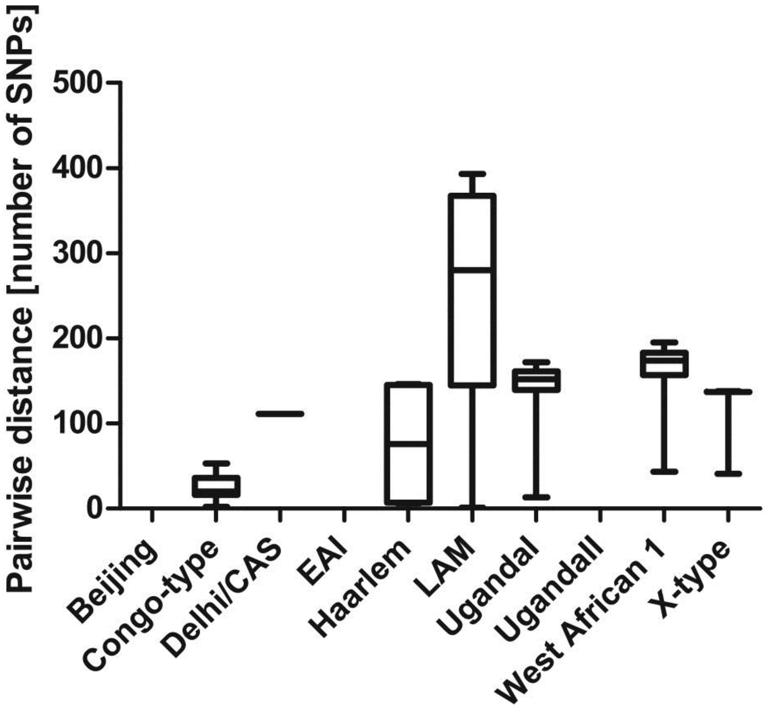

Technical Appendix Figure 2. Intralineage pairwise distance comparison of the population of the Republic of the Congo *Mycobacterium tuberculosis* study. Box and whisker plot showing the minimum, median, maximum, 25th and 75th percentiles of pairwise distances of the lineages of the study population (number of isolates per lineage/sublineage: Beijing n = 1, Congo type, n = 26; Delhi/CAS, n = 2; EAI, n = 1; Haarlem, n = 4; LAM, n = 12; Uganda I, n = 7; Uganda II, n = 1; West African 1, n = 6; X type, n = 3). CAS, Central Asian strain; EAI, East African Indian; LAM, Latin American Mediterranean.
